# Complete Genome Sequence of A388, an Antibiotic-Resistant Acinetobacter baumannii Global Clone 1 Isolate from Greece

**DOI:** 10.1128/MRA.00971-19

**Published:** 2019-10-10

**Authors:** Mohammad Hamidian, Ryan R. Wick, Louise M. Judd, Kathryn E. Holt, Ruth M. Hall

**Affiliations:** a School of Life and Environmental Sciences, The University of Sydney, Sydney, NSW, Australia; b The ithree Institute, University of Technology Sydney, Ultimo, NSW, Australia; c Department of Infectious Diseases, Central Clinical School, Monash University, Melbourne, Victoria, Australia; d London School of Hygiene & Tropical Medicine, London, United Kingdom; University of Arizona

## Abstract

Acinetobacter baumannii isolate A388, recovered in Greece in 2002, represents a distinct antibiotic-resistant lineage of global clone 1 (GC1) producing the OXA-58 carbapenemase. We present the complete 4.332-Mbp genome sequence (chromosome plus 1 plasmid), generated by combining long (MinION) and short (Illumina HiSeq) read sequencing data.

## ANNOUNCEMENT

The Acinetobacter baumannii isolate A388, recovered in 2002 at a hospital in Ioannina, Greece, during a survey of carbapenem resistance in Europe (1), was obtained from the collection of Kevin Towner (Nottingham University Hospital, United Kingdom). A388 was previously shown to be representative of a distinct lineage of global clone 1 (GC1) designated sequence group 6 (SG6) (2, 3). It belongs to sequence type 1 (ST1) (Institut Pasteur multilocus sequence typing scheme) and ST439 (Oxford scheme; formerly ST248) and carries the KL20 gene cluster at the capsule biosynthesis locus and OCL4 at the directing synthesis locus of the outer core of lipooligosaccharide (4, 5). A388 is resistant to the frontline carbapenem antibiotics imipenem and meropenem via carriage of the *oxa58* gene (4, 6). It also carries IS*Aba125* rather than IS*Aba1* upstream of the *ampC* gene, increasing the expression of AmpC and giving rise to third-generation cephalosporin resistance (7). Moreover, the A388 lineage appears to be the source of the IS*Aba125*::*ampC* configuration found in the global clone 2 isolate ACICU from Italy (7). A388 is susceptible to fluoroquinolones but includes the *aphA6* aminoglycoside resistance gene in transposon Tn*aphA6* (4) and genes *aacA4*, *aacC1*, and *aphA1* in the AbaR30 resistance island (8), conferring resistance to most aminoglycosides (amikacin, gentamicin, kanamycin, neomycin, netilmicin, and tobramycin). AbaR30 is located in the *comM* gene and also includes *sul1* and *tetA*(A) genes for resistance to older antibiotics (sulfonamides and tetracycline, respectively).

Whole-cell DNA was prepared from cells grown at 37°C in LB inoculated from a frozen stock derived from a single colony, quality controlled for length, and quantified as described previously (9, 10). The DNA was subjected to library preparation, barcoding, and MinION (Oxford Nanopore Technologies) sequencing as described in detail elsewhere (10). Reads were base called using Albacore v1.2.5 and trimmed and demultiplexed with Porechop v0.2.1, as described in detail elsewhere (10). A total of 23,066 reads were obtained with an *N*_50_ length of 18.1 kbp and 50-fold coverage. The MinION reads (251.9 Mbp; SRA accession number SRR9821831) were retrieved from the raw data and subsampled for length and quality using Filtlong v0.1.0, as described in detail elsewhere (10), resulting in 17,549 reads (200 Mbp), which were used for assembly. These MinION reads were combined with available Illumina HiSeq data (SRA accession number ERX087515; coverage, 98-fold) reported previously (4) using Unicycler v0.4.4 (11) with default parameters.

The assembly consisted of the chromosome, a contiguous circular sequence of 3,999,012 bp (G+C content of 39.26%), and one 33,036-bp plasmid, pA388. However, the IS26* (IS*26*-v1) in AbaR30 has caused a chromosomal inversion splitting AbaR30 into two parts (8), namely, AbaR30a (bases 244861 to 290116) and AbaR30b (bases 3763191 to 3781931). Protein-coding, rRNA, and tRNA genes were initially annotated automatically using Prokka v1.12 (12), and annotations of the KL20 and OCL4 polysaccharide biosynthesis loci were refined using current gene nomenclature (5, 13). Annotation of the antibiotic resistance regions and the plasmid were also refined manually. Transposons and *dif* modules and p*dif* sites (14) in the plasmid were identified manually and annotated. Tn*aphA6* is in the chromosome (bases 2265632 to 2268703), and pA388 carries the *oxa58* gene in a *dif* module and the *aphA1* gene in transposon Tn*4352*. In addition to the IS in AbaR30a and AbaR30b, 6 copies of IS*Aba1* and 11 of IS*Aba125*, as well as 12 of IS*Aba12* and single copies of IS*Aba2* and IS*26*, were detected in the chromosome using ISfinder (https://isfinder.biotoul.fr/). Two potential integrated phage genomes of 23.5 kbp (bases 614654 to 638202) and 56 kbp (bases 1353058 to 1409116) were identified using PHASTER (15). All identified features are annotated in the released GenBank files.

The genome sequence of A388 will underpin studies of the origin and evolution of the unique GC1 lineage found in hospitals in the Eastern Mediterranean.

### Data availability.

The complete genome sequence has been deposited in DDBJ/ENA/GenBank under the accession numbers CP024418 (chromosome) and CP024419 (pA388). The versions described in this paper are the first versions, CP024418.1 and CP024419.1. The MinION reads have been deposited in the SRA under the accession number SRR9821831. Illumina HiSeq data are available under accession number ERR110081 (SRA accession number ERX087515).
